# Recent advances in understanding and managing urolithiasis

**DOI:** 10.12688/f1000research.9570.1

**Published:** 2016-11-08

**Authors:** Walter L. Strohmaier

**Affiliations:** 1 Department of Urology and Paediatric Urology, Regiomed-Kliniken, Coburg, Germany; 2 Academic Hospital of the University of Split, Split, Croatia

**Keywords:** endourology, stone formation, calcium oxalate, extracorporeal shockwave lithotripsy, urolithiasis

## Abstract

During the last few years, there has been relevant progress in both understanding and managing urolithiasis. Our knowledge of stone formation has changed; although the importance of urine biochemistry was questioned by several investigators years ago, the decisive role of cellular processes (induced by oxidative stress) and the renal papilla has only recently been generally accepted as the most important step in stone formation. For calcium oxalate urolithiasis, the formation of papillary calcifications plays a key role and is of prognostic relevance. Further research has to concentrate on these aspects of preventing urolithiasis. Stone prevention (metaphylaxis) is a major issue when considering the burden it places on healthcare systems. An effective metaphylaxis could lower the cost of stone therapy significantly. For uric acid urolithiasis, so far there is only preliminary information available showing that papillary plaques are not as important as they are in calcium oxalate urolithiasis. Concerning stone management, endourology has improved stone therapy significantly during the last few years. Morbidity decreased and success (stone-free) rates increased. Therefore, the indications for extracorporeal shockwave lithotripsy (ESWL) narrowed. ESWL, however, still has its place in stone therapy. There is not one single treatment modality that is equally effective for all situations. It is important to observe the differential indications for different stones depending on size, localization, and composition.

## Introduction

Urolithiasis places a significant economic burden on the healthcare system, especially in industrialized countries where, owing to changes in lifestyle and diet, the incidence of stone disease has steadily increased over the last several decades; unfortunately, it will probably continue to increase for a number of reasons, one of which is global warming ^1, 2^. Therefore, the costs of medically and surgically treating as well as diagnosing stones will also rise significantly ^3^. This highlights the enormous importance of urolithiasis for our healthcare systems. During the last few years, there has been relevant progress in both understanding and managing this disease.

### Understanding urolithiasis

Although first postulated almost 80 years ago ^4^, the decisive role of cellular processes and the renal papilla has only recently been generally accepted as the most important step in stone formation.

### Managing urolithiasis

Endourologic techniques were introduced into urolithiasis therapy about 40 years ago. Nevertheless, they have been generally promoted with extending the indications since the beginning of the 21st century. The main reason for this is improvements in the endoscopic instruments. This allowed higher stone-free rates on one hand and lower complication rates on the other. At this time, all types of stones in any localization may be treated by endoscopic procedures. In consequence, the application of extracorporeal shockwave lithotripsy (ESWL) decreased. Nevertheless, ESWL is still widely used in stone therapy. According to the EAU guidelines ^5^, ESWL can be used in nearly all stone locations. Unfavorable factors are shockwave-resistant stones (whewellite, brushite, or cystine), a steep infundibular-pelvic angle, a long lower pole calyx (>10 mm), and a narrow infundibulum (<5 mm).

## Understanding urolithiasis

Today, urolithiasis is an economic challenge for all healthcare systems ^6^. Because of changes in lifestyle, dietary habits, and treatment modalities, its incidence and prevalence rose significantly over the last few decades ^1^. One can expect that the frequency of urolithiasis will rise even more (by 7–10%) owing to global warming, since stone disease is encountered more frequently in hot regions ^3^.

A prerequisite for urinary stone formation is the supersaturation of the urine with stone-forming substances like calcium, oxalate, phosphate, and uric acid. Therefore, until now, prevention strategies have been directed towards changes in urine biochemistry ^7^. Although the importance of urine biochemistry was questioned by several investigators years ago, it was only recently widely accepted that urine biochemistry cannot explain stone formation exclusively ^8^. Many individuals with urinary supersaturation never form stones ^9^.

With the exception of some very rare types of stone disease (e.g. primary hyperoxaluria and cystinuria), for physicochemical reasons crystals cannot grow into stones within the renal tubules, as the transit time is too short (free particle theory). For stone growth, crystals have to be fixed to tubular cells or renal tissue (fixed particle theory ^10^). A prerequisite for fixation, however, is a lesion of the tissue or cells ^11^. Picking up these theories, Robertson recently developed a computer model (NEPHROSIM) that attempts to improve the understanding of reabsorption and secretion processes in the renal tubules and their relevance for the initial processes of calcium oxalate (CaOx) stone formation ^12^.

Although these facts have been known for several decades, they came into focus and were generally accepted only recently. Especially for CaOx urolithiasis, the most common type, stone formation starts with the formation of plaques developing in the basement membrane of the thin loop of Henle ^13^. These Randall’s plaques consist of apatite and organic material (glycoproteins, glycosaminoglycans, and lipids). Randall’s plaques are the nidus for CaOx stone formation (papillary calcifications). In cases of urine supersaturation with calcium and oxalate, CaOx crystals adhere and overgrow the apatite plaques. Therefore, it can be stated that there are two prerequisites for CaOx stone formation: 1. cellular injury and apatite plaque formation and 2. urine supersaturation with calcium and oxalate.

Why these plaques form is a complex phenomenon and not fully understood. However, there is increasing evidence that renal tubular cell damage and localized inflammation play an important role ^14^. Apart from these plaques, Randall ^15^ described a second type of papillary lesion (papillary lesion type II), which is an intratubular calcification in the distal part of Bellini’s duct. Today, they are called Randall’s plugs. They are predominantly found in patients with higher supersaturation of the urine (e.g. primary hyperoxaluria and primary hyperparathyroidism). However, they are also encountered in idiopathic CaOx stone formers ^16^.

### Calcium oxalate urolithiasis

We were interested in how frequently papillary calcifications can be encountered in patients with idiopathic CaOx urolithiasis (iCaOxU), the most frequent stone type, and whether the assessment of their extent may be used for predicting the risk of recurrence.

**Figure 1.**
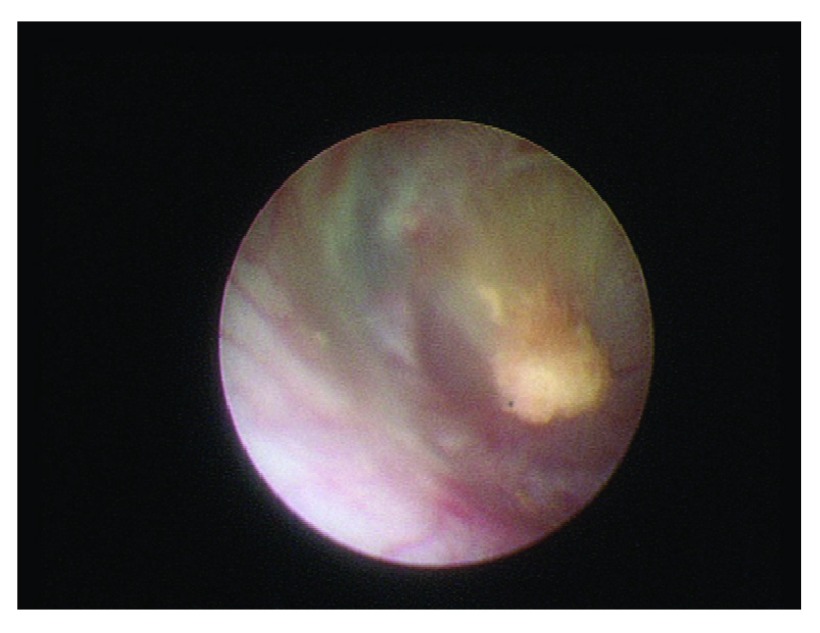
Renal papilla with high-grade calcifications in a patient with idiopathic calcium oxalate urolithiasis.

We studied 100 patients with iCaOxU undergoing stone treatment by flexible endourologic instruments ^17^. The renal papillae were examined and counted. In addition, the extent of plaques was determined ( Figure 1). The so-called calcification index (CI) was calculated: sum of the number of renal papillae multiplied with the grade of calcifications (1–3) multiplied with the number of papillae with calcifications divided by the total number of papillae. Also, a metabolic assessment was done. The CI correlated significantly (r=0.37; p=0.012) with the number of stone episodes in the patients’ histories.

Concerning the metabolic parameters, only citrate (r=0.51; p=0.002) correlated significantly with the number of stone episodes. This is a paradox we can’t explain, as citrate is an inhibitor of CaOx stone formation. These findings highlight the importance of Randall’s plaques in the pathogenesis of iCaOxU. Moreover, the assessment of papillary plaques or calcifications by means of endourology and calculating the CI is a reliable prognostic factor in contrast to conventional metabolic (biochemical) parameters.

The next question to be discussed is how these papillary plaques form. The whole complex is not completely understood so far; however, there is increasing evidence that renal tubular cell damage and localized inflammation play an important role ^14, 18^. There are two potential ways renal epithelial injury may occur ^11^: firstly, CaOx and calcium phosphate crystals cause cellular damage, and, secondly, crystals adhere and grow on injured renal epithelial cells. For both potential pathways, oxidative stress and lipid peroxidation are important factors. Some studies showed that parameters of oxidative stress and lipid peroxidation are increased in renal stone formers ^14, 19– 22^.

Experimentally, medications protecting against oxidative stress (e.g. calcium antagonists, N-acetyl-cysteine, and phytopharmaceuticals) could interfere with these mechanisms and lower urinary stone formation ^1, 23– 30^. These observations could open up new options in renal stone prevention and metaphylaxis (secondary prevention) ^31^. Standardized preparations like Canephron N containing centaury, lovage, and rosemary exhibit not only anti-oxidative and nephroprotective but also diuretic and anti-inflammatory effects. This unique combination of antiurolithiatic effects is very promising ^32, 33^. However, this has to be validated by randomized studies. Further research has to concentrate on these aspects of urolithiasis prevention. Stone prevention (metaphylaxis) is a major issue considering the burden it places on healthcare systems. Metaphylaxis is not only medically but also economically effective. An effective metaphylaxis could lower the cost of stone therapy significantly ^34, 35^.

### Uric acid urolithiasis

Since uric acid (UA) stones are unusually common in our region (Upper Franconia, Germany, 20–25% of all stone formers) ^36^, we investigated the meaning and the importance of papillary plaques in this type of urolithiasis. So far, there are only very limited data existing in the literature. Viers *et al*. ^37^ published a series of 23 patients with stones containing UA. However, only four had pure UA stones. The vast majority was mixed with CaOx. Until now, we have examined 30 patients suffering from pure UA stones. The study design was as outlined above for iCaOxU. Our preliminary data showed that – contrary to iCaOx (7.7 ± 7.9) – the CI was significantly lower in UA stones (5.04 ± 4.39). Nevertheless, the number of stone episodes or recurrence rate was higher in UA stones. There was no correlation between the CI and recurrence rate. Regarding the biochemical metabolic parameters, blood calcium correlated positively and urine pH and volume negatively with the recurrence rate.

UA plaques of the renal papillae obviously are not of such importance in the pathogenesis of stone formation as they are in iCaOxU. According to our preliminary results, they do not correlate with the number of recurrences. Therefore, they may not be used to predict the risk of recurrence. However, these first results should be confirmed in larger numbers of UA stone patients. Our observations again demonstrate that urolithiasis is a very complex phenomenon and that there are different pathways in stone formation for the different types of stones.

## Managing urolithiasis

Since the beginning of the 21st century, the general acceptance and the dissemination of endoscopic therapy modalities rose dramatically ^38– 41^. The word “endourology” goes back to Arthur Smith. It means a “closed, controlled manipulation in the genitourinary tract”. In the field of renal and ureteral stones, endourologic treatment modalities include ureterorenoscopy (URS), laparoscopic ureterolithotomy, percutaneous nephrolithotomy (PCNL), and laparoscopic pyelolithotomy. The main reason for this dramatic rise in the use of these procedures is technical improvements in the instruments. These newer instruments allow for higher success rates and reduced morbidity.

Nevertheless, ESWL is still widely used for treating both ureteral and renal stones. The number of ESWL treatments, however, has decreased during the last few decades. In contrast, endourologic procedures were used more frequently ^39, 42– 44^. At this time, urinary stones of all types and localizations can be treated by endoscopic modalities with similar stone-free rates and morbidity in comparison to ESWL.

### Management of renal stones

ESWL is indicated preferentially in renal stones up to 2 cm in diameter when located in the renal pelvis and upper and middle calyces. In these cases, stone-free rates from 80–100% can be achieved sometimes; however, it requires multiple sessions. Complication rates range from 0–20% ^5^.

Lower pole stones do not respond so well, especially in the case of unfavorable factors for ESWL (shockwave-resistant stones [whewellite, brushite, and cystine], steep infundibular-pelvic angle, long lower pole [>10 mm], and narrow infundibulum [<5 mm]). Those stones should be treated preferentially by endourologic modalities ^5, 45, 46^.

**Figure 2.**
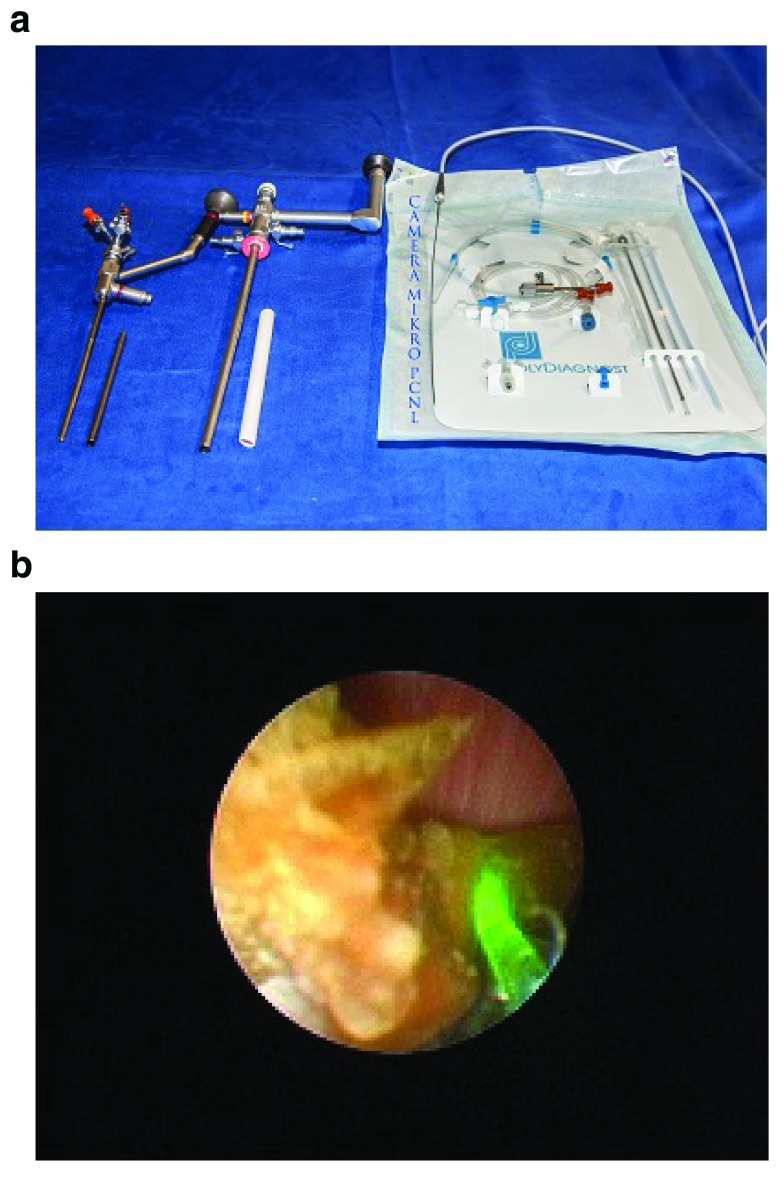
**a**. Mini, standard, and micro percutaneous nephrolithotomy (PCNL) instruments. **b**. Stone disintegration using a holmium laser during mini percutaneous nephrolithotomy (PCNL).

Various forms of PCNL (conventional, mini, ultra-mini, and micro PCNL) are available for treating all renal stones ( Figure 2a and 2b). The range of the diameters of these instruments varies between 5 (micro PCNL) and 34 F (large standard PCNL) ^16, 47^. Although there is some evidence that smaller instruments cause less trauma, this has not really been proven ^5^. On the other hand, operating time increases with decreasing diameter of the instrument. Further studies are required to establish the definitive role of miniaturized instruments. In the meantime, it seems wise to adapt the diameter of the instruments to the size of the stone.

The overall complication rates for PCNL range from 0–30%. Concerning stone-free rates, the diameter, composition, and localization of the stones have to be considered. Stone-free rates range from 100% in small to about 60% in complex (e.g. staghorn) stones ^5^.

Retrograde intrarenal surgery (RIRS) using flexible ureterorenoscopes can be used to manage especially small stones. According to size, composition, and localization of the stone, stone-free rates vary from 65–100%. Complications occur in 0–20% ^5, 45, 46, 48, 49^.

In a select few cases, laparoscopic stone removal is another option (treatment failure of other modalities and concomitant anomalies such as pyelo-ureteral junction obstruction and obstructing cysts). Open surgery is extremely rare today ^5, 45, 46^.

### Management of ureteral stones

ESWL may be used for all stones in the ureter. The stone-free rates range from 85–100%. Complication rates range from 0–20%. However, large distal stones should be treated preferentially by endourologic therapy, as the stone-free rates are higher ^5, 45, 46^.

**Figure 3.**
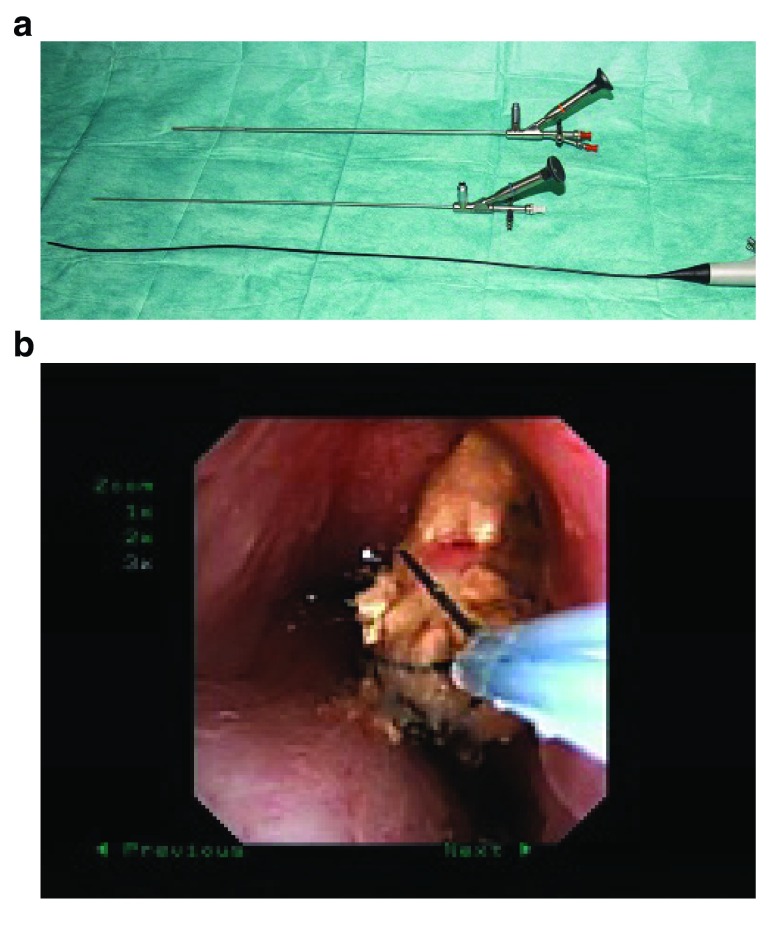
**a**. Semirigid and flexible instruments for ureterorenoscopy. **b**. Stone extraction from a renal calyx using a flexible ureterorenoscope and a tipless basket.

For endoscopic therapy of ureteral stones, different types of ureteroscopes (semirigid and flexible) are available ( Figure 3a and 3b). To reduce the trauma, small instruments with tip diameters <8 F should be used. The stone-free rates range from 85–100% and the complication rates between 0 and 20% ^5, 45, 46^. In unusual situations, laparoscopic surgery may be indicated (treatment failures of endourologic modalities and extremely large stones). Open surgery is almost never required today ^5, 45, 46^.

### Stone fragmentation

In many situations, in the kidney and the ureter as well, stones are too large to be extracted completely. Therefore, they have to be disintegrated (fragmented). For this purpose, a number of approaches are available, such as laser, ultrasound, and pneumatic. The gold standard is the holmium laser, as it is highly effective and can be used with flexible devices. When treating larger stones with semirigid and rigid instruments (URS and PCNL), pneumatic and ultrasound lithotripters can be used, as they reduce operating time ^50^.

In summary, endourology has improved stone therapy significantly over the last several years. Morbidity decreased and success (stone-free) rates increased. Therefore, the indications for ESWL narrowed. However, when used in the broad field and looked at carefully, the re-intervention rate for residual fragments after URS was about 40%. This is similar to that of ESWL ^51, 52^. Potentially, these differences in the success rates of URS are because of different techniques (e.g. using access sheaths and flexible instruments or not, or removing the stone fragments using baskets or dusting the stone). In the end, it could be that URS may not be quite as effective as we like to believe ^53^. ESWL still has its place in stone therapy. There is not one single treatment modality that is equally effective for all situations. It is important to observe the differential indications as outlined above.

## Future outlook

Some questions remain to be answered in the future. Will further miniaturization of the instruments really be less traumatic and give the same results? Could stone fragmentation be improved (e.g. by more effective lasers)? Another issue is economy: flexible ureterorenoscopes, while being versatile and less traumatic, are expensive and not very durable. Their further dissemination is dependent on economic factors. Will single-use instruments help ^54^? Recently, robotic support has been introduced to endourologic stone therapy. However, so far, there are limited advantages ^55^.

## Abbreviations

CaOx, calcium oxalate; CI, calcification index; ESWL, extracorporeal shockwave lithotripsy; iCaOxU, idiopathic calcium oxalate urolithiasis; PCNL, percutaneous nephrolithotomy; RIRS, retrograde intrarenal surgery (RIRS); UA, uric acid; URS, ureterorenoscopy.
